# 830 Burn Injury Outcomes in Organ Transplant Patients: A Review of Institutional and National Data

**DOI:** 10.1093/jbcr/iraf019.361

**Published:** 2025-04-01

**Authors:** Hilary Liu, Mare Kaulakis, José Arellano, Christopher Fedor, Garth Elias, Alain Corcos, Jenny Ziembicki, Francesco Egro

**Affiliations:** University of Pittsburgh Medical Center; University of Pittsburgh School of Medicine; University of Pittsburgh Medical Center; University of Pittsburgh School of Medicine; University of Pittsburgh Medical Center; University of Pittsburgh Medical Center; University of Pittsburgh Medical Center; University of Pittsburgh Medical Center

## Abstract

**Introduction:**

Transplant patients often face immunosuppression, pre-existing conditions, and poor circulation, increasing their risk of infection and wound healing complications. This study evaluates the outcomes of burn patients with a history of organ transplants at a single institution and on a national scale.

**Methods:**

We conducted a retrospective cohort study on transplant patients treated for burn injuries at an ABA-verified burn center from January 2012 to October 2023. Additionally, data from the ABA Burn Care Quality Platform (BCQP) from January 2013 to December 2015 was extracted for transplant patients. The data collected included demographics, transplant details, injury characteristics, surgical interventions, and complications.

**Results:**

11 patients (100% male; mean age of 54.6±20.4 years) at our institution had prior organ transplants. All patients sustained thermal burns, with a mean total body surface area (TBSA) of 7.8±12.4%. Most patients had undergone kidney (n=5; 45.5%) or liver (n=4; 36.4%) transplants. The average time from transplant to burn injury was 7.9±7.0 years. Five patients (45.5%) did not require surgical intervention, while six patients (54.5%) underwent surgical burn excision and grafting. Four patients (36.4%) experienced infection requiring intravenous antibiotics, and two (18.2%) experienced partial graft loss. Unfortunately, four patients (36.4%) died due to non-burn-related complications.

47 patients (76.6% male, 23.4% female; mean age of 55.8±15.2 years) in the BCQP had prior organ transplants. Flame injuries (n=11; 23.4%) and scalds (n=9; 19.15%) were the most common causes of burn injury, and the average TBSA affected was 7.6±11.2%. Most patients (n=33; 70.2%) required burn excision and grafting. Interestingly, there were no reported graft loss or infections. One patient (2.1%) died during their hospital stay.

**Conclusions:**

Burn patients with a history of organ transplants face significant challenges, including a higher risk of infection and wound healing complications. Tailored management strategies are crucial for improving outcomes in this vulnerable population.

**Applicability of Research to Practice:**

This study underscores the need for tailored management strategies in burn care for transplant patients, who face increased risks of infection and wound healing complications. The findings can guide healthcare providers in developing evidence-based protocols to optimize treatment and improve outcomes for this vulnerable population.

**Funding for the Study:**

N/A

